# Deep phenotyping of suicidal ideation after discharge from psychiatric inpatient care: study protocol for an interdisciplinary, multicentre prospective observational study in Psychiatric University Hospitals

**DOI:** 10.1136/bmjopen-2025-111273

**Published:** 2026-02-06

**Authors:** Anna Monn, Stephanie Homan, Jacopo Mocellin, Schwarna Maria Raja, Lara Kirchhofer, Vivienne Walser, Eyal Liron Dolev, Marcia Nißen, Tobias Kowatsch, Guido Seiler, Katharina Schultebraucks, Sebastian Olbrich, Birgit Kleim

**Affiliations:** 1 Department of Adult Psychiatry and Psychotherapy, Psychiatric University Hospital Zurich, Zurich, Switzerland; 2 Department of Experimental Psychopathology and Psychotherapy, University of Zurich, Zurich, Switzerland; 3 Healthy Longevity Center, University of Zurich, Zurich, Switzerland; 4 German Department, University of Zurich, Zurich, Switzerland; 5 Centre for Digital Health Interventions, ETH Zurich, Zurich, Switzerland; 6 School of Medicine, University of St Gallen, St. Gallen, Switzerland; 7 Institute for Implementation Science in Health Care, University of Zurich, Zurich, Switzerland; 8 Zurich Center for Linguistics, University of Zurich, Zurich, Switzerland; 9 Department of Psychiatry, New York University Grossman School of Medicine, New York, New York, USA; 10 Division of Healthcare Delivery Science, New York University Grossman School of Medicine, New York, New York, USA

**Keywords:** Suicide & self-harm, Observational Study, Machine Learning, Digital Technology, Hospital to Home Transition

## Abstract

**Abstract:**

**Introduction:**

Suicidal thoughts and behaviours (STB) are a critical public health concern, with 700 000 deaths by suicide each year. The period immediately following hospital discharge is associated with an elevated risk for suicide. Monitoring suicidal ideations throughout this period is therefore critical. However, its highly dynamic nature limits the utility of traditional risk assessments through infrequent outpatient visits. Recent advancements in ambulatory assessments and multimodal predictive approaches offer a promising new avenue. Hence, the present study aims to examine how psychological, linguistic, neurobiological and smartphone-based characteristics relate to suicidal ideation and to improve STB monitoring through a deep phenotyping approach.

**Methods and analysis:**

In this interdisciplinary, multicentre, prospective observational study, we plan to recruit a total of 200 inpatients with current and/or past STB. The study comprises the following components: (1) a baseline assessment, conducted while participants are still in the hospital. This includes interviews, an electroencephalography recording, a video-recorded verbal task and self-report questionnaires; (2) data collection through a smartphone application during the first 4 weeks after hospital discharge with two active collection weeks of five daily ecological momentary assessments and two 1 min video diaries every other day, as well as smartphone passive sensing for 28 consecutive days and (3) two follow-up assessments, 4 weeks and 3 months after discharge. The primary outcome is self-reported suicidal ideation after hospital discharge.

**Ethics and dissemination:**

The Ethics Committee of the Faculty of Arts and Social Sciences of the University of Zurich, Switzerland, approved the study for the Zurich and Basel sites (Ref: 22.09.19). Approval for the New York Site was granted by the Institutional Review Board of NYU Langone Health (i23-00366). Study findings will be disseminated via peer-reviewed, open-access publications, conference presentations, patient and public events, and dedicated social media outlets.

**Trial registration number:**

CRSII5_205913.

**STRENGTHS AND LIMITATIONS OF THIS STUDY**

- This study’s strength lies in the integration of diverse data sources (psychological, linguistic, neurobiological and digital) for deep phenotyping.
- The high-frequency real-time assessments (ecological momentary assessments and video diaries) allow for capturing suicidal ideation dynamics.
- Potential limitations related to the study include that intensive assessments may lead to dropout or reactivity effects, as well as biases due to variability in participants’ digital literacy or smartphone models.
- Potential limitations related to study design include the observational nature, which limits causal inference regarding risk factors for suicidal ideation.

## Introduction

 Suicidal thoughts and behaviours (STB) represent a critical public health concern, with more than 700 000 suicide deaths annually worldwide and rising suicide rates in countries such as England, Wales and the USA.^1^,^4^ Still, current predictive models have shown limited utility, and effective interventions remain scarce.^5^,^7^ The Integrated Motivational-Volitional (IMV) Model of Suicidal Behaviour conceptualises the emergence of STB as a process unfolding across three distinct phases: the premotivational phase encompassing the biopsychosocial context for STB, the motivational phase where suicidal ideation develops from defeat and entrapment, and the volitional phase describing suicidal enactment, with multiple moderators influencing transitions between these phases.^8^ The complex interplay of proximal and distal risk factors outlined in the IMV model highlights the need for a deep phenotyping approach that integrates multiple data sources to effectively anticipate trajectories of STB.^8^,^10^ This may include incorporating psychological, clinical, social, neurobiological and digital signals. One context in which such an approach is particularly critical is the period immediately following discharge from a psychiatric hospital, as it is associated with a markedly elevated suicide risk, with rates up to 100 times the global suicide rate, regardless of the reason for admission, and up to 200 times higher when patients were hospitalised due to STB.^11^ Suicidal ideations, including thoughts of or the desire to die, are a major risk factor for suicide attempts and deaths, underscoring its relevance as a clinically actionable monitoring target in the postdischarge period.^7^ However, the highly dynamic nature of suicidal ideations, often fluctuating over a single day, limits the utility of infrequent, self-reported clinician assessments.^12^,^14^ To address this challenge, recent advancements in digital phenotyping—defined as ‘the moment-by-moment quantification of the individual-level human phenotype in situ using data from personal digital devices’^15^ such as passive sensing data and data from ecological momentary assessments (EMA)—offer promising opportunities for more continuous and ecologically valid monitoring of suicidal ideation.^16 17^ These techniques may draw from various data sources (eg, behavioural, psychological, environmental and social phenotyping) and collection methods, which are typically divided into active and passive data collection methods. Active data collection requires active user input, for example, in the form of EMA, where suicidal ideation and related constructs are assessed via repeated smartphone prompts throughout the day, or through video diaries, from which facial, speech and linguistic features can be derived.^1317^,^20^ Passive data collection, in contrast, occurs unobtrusively, for example, via smartphone sensors (eg, call logs or step counts).^17^ Several studies have investigated how within-person and between-person dynamics of EMA-assessed suicide-related constructs are associated with near-term and long-term STB during the postdischarge period, with some showing promising results.^21^,^24^ Conversely, the extent to which passive data collection methods offer incremental predictive value has yet to be determined.^25^

Addressing these gaps, the present study will combine both active and passive data collection methods. It will extend beyond previous EMA-passive-sensing studies by incorporating objective pre-discharge neurobiological markers derived from resting-state electroencephalography (EEG), as well as video-based assessments to extract digital biomarkers and language-based features. Integrating these complementary data streams could represent a promising strategy for enhancing the accuracy and clinical utility of STB monitoring strategies in the postdischarge period.^26^ Hence, this multicentre, observational study, MULTICAST-PREDICT (A Multidisciplinary Approach to Predict and Treat Suicidality), has two main goals: (1) to examine how key baseline variables (ie, language-based and speech-based, demographic, clinical-psychological and neurobiological characteristics) and daily diary assessments capturing speech, clinical and behavioural correlates are associated with suicidal ideation after hospital discharge and (2) to study suicidal ideation in at-risk individuals using a deep-phenotyping approach, combining the available data sources.

According to the main research aims, we stated the following primary hypotheses, each targeting a distinct domain or modality of investigation:

(H1) Individual trajectories of digital phenotyping data, including both EMA responses and smartphone passive sensing data, during the 4-week postdischarge period are associated with suicidal ideation 4 weeks after hospital discharge.
(H2) Syntactic patterns, specifically less exploitation of information structural means and less syntactic variance, are associated with suicidal ideation 4 weeks after hospital discharge.
(H3) Baseline resting-state EEG signatures, indexed by impaired EEG vigilance regulation, increased alpha functional connectivity, and frontal alpha asymmetry, are associated with suicidal ideation 4 weeks after hospital discharge.
(H4) Advanced machine learning approaches that integrate multimodal baseline features, including digital biomarkers (ie, visual and auditory markers), language, demographic, clinical and neurobiological data, will significantly enhance the prediction accuracy of suicidal ideation 4 weeks postdischarge. Specifically, the inclusion of digital biomarkers and language-based features will yield a measurable enhancement in model performance beyond other modalities alone.

In addition to these primary hypotheses, we will also explore the scalability of our methods to better capture real-time dynamic fluctuations of STB. Specifically, if manually coded reduced use of information structural means and reduced syntactic complexity are consistent markers of suicidal ideation, we will examine whether these can be reliably identified using large language models (LLMs). Furthermore, we will explore whether digital phenotyping data, collected both passively via smartphone sensors (‘smartphone passive sensing’) and actively via EMA, can predict subsequent EMA entries within individuals. These exploratory components could help lay the groundwork for developing semi or fully automated tools capable of detecting linguistic, psychological and behavioural risk markers in naturalistic settings. Such tools would enable scalable monitoring and may ultimately support timely intervention strategies.

## Methods and analysis

We adhered, where applicable, to the Strengthening the Reporting of Observational Studies in Epidemiology (STROBE) guidelines for the reporting of this protocol of an observational, longitudinal study.^27^

### Patient and public involvement

This study is based on the feasibility study SIMON (Suicidal Ideation Monitoring).^23^ As part of that study, participants provided feedback on EMA assessments, the mobile phone application and overall study participation. Further, we conducted a stakeholder interview with a former study participant, clinicians (ie, an attending, a resident and a psychologist), and a nurse to gather insight into the daily clinical needs and wishes for digital interventions. The insights gained served as the basis for the further development of this study’s design and monitoring application.

### Design

**Figure 1:**
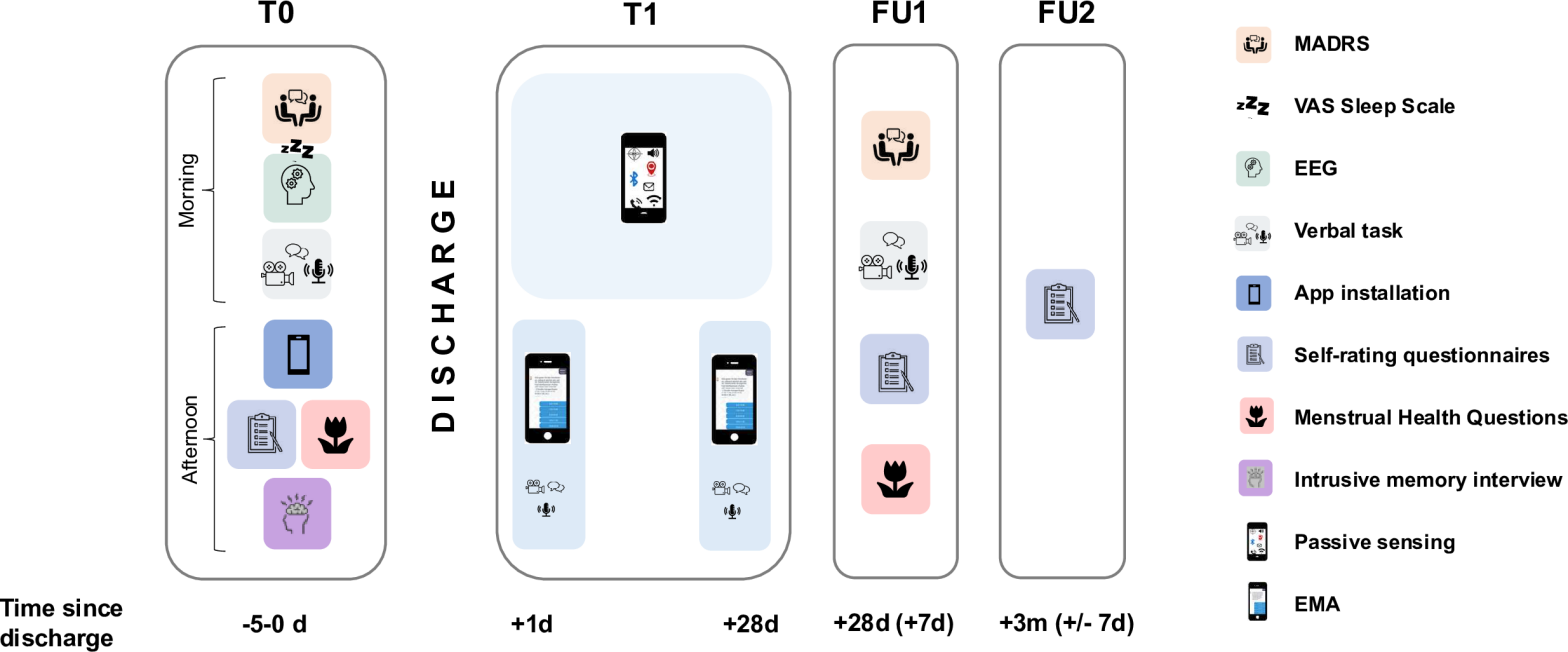
Study design of MULTICAST-PREDICT. (**T0**): The baseline assessment is conducted 0–5 days prior to hospital discharge and consists of a morning and an afternoon session, each lasting approximately 1.5–2 hours. During the morning session, participants complete the Montgomery-Asberg Depression Rating Scale (MADRS), a visual analogue scale (VAS) for sleep quality, a resting-state electroencephalography (EEG) recording, and a video-recorded verbal task with simultaneous EEG acquisition. The afternoon session includes the installation of a smartphone application application and self-report questionnaires, including an additional set of menstrual-health related questions. Additionally, participants with a history of suicide attempt(s) complete the Intrusive Memory Interview. (**T1**): Data collection via the smartphone application starts 1 day after hospital discharge. This involves 28 days of continuous smartphone passive sensing as well as two burst weeks—separated by a 14-day interval—of five daily ecological momentary assessments (EMA) and video diaries every other day. (FU1): Follow-up 1 occurs 28–35 days after discharge and features the MADRS, the video-recorded verbal task and self-report questionnaires. Additionally, participants provide feedback on the monitoring app. (FU2): FU 2 occurs 3 months (±7 days) after discharge and involves self-report questionnaires. Both follow-up assessments may take place either in person or via video call. Abbreviations: d, day; EEG, electroencephalogram; EMA, ecological momentary assessment; MADRS, Montgomery-Asberg Depression Rating Scale; m, month; MULTICAST-PREDICT, A Multidisciplinary Approach to Predict and Treat Suicidality; T0, baseline assessment; T1, smartphone active and passive sensing phase; VAS, Visual Analogue Scale; w, week.

This interdisciplinary, multicentre prospective observational study employs an uncontrolled, within-group design. The study comprises a baseline assessment, followed by two 7-day weeks of active data collection postdischarge (separated by a 14-day interval) involving daily EMA prompts and video diaries every other day. Additionally, the monitoring application collects 28 days of smartphone passive sensing data. Follow-up assessments are conducted 4 weeks and 3 months post-discharge (see figure 1).

### Eligibility criteria

The primary inclusion criterion is the presence of current and/or past STB, encompassing passive and active thoughts of suicide as well as suicide attempts with either explicit or implicit intent to die. This comprehensive criterion was purposefully selected to capture the full spectrum of severity associated with STB.^28^ Additional inclusion criteria comprise current inpatient psychiatric treatment, sufficient proficiency in German, age between 18 and 65 years, and regular use of a smartphone.

Exclusion criteria include the presence of active psychotic symptoms, cognitive impairment or a scheduled electroconvulsive therapy session within 3 months preceding the baseline assessment. Individuals who share their smartphone with others, are active military personnel or are pregnant or breastfeeding at baseline are also excluded.

### Recruitment

A total of 200 participants will be recruited from three centres: the University Hospital of Psychiatry Zurich, Switzerland (PUK, n=100), the University Psychiatric Clinics Basel, Switzerland (UPK, n=50) and the Psychiatric Unit of New York University Langone Health, United States of America (NYU, n=50). Data collection has started in October 2023 and is expected to be concluded by mid-2026. In each centre, recruitment involves a preliminary screening across all hospital admission for primary inclusion criteria, either through a designated study recruitment team (PUK, NYU) or via direct access to personal health records (UPK). Next, research coordinators consult with the treating clinical staff for detailed screening. If a patient meets the eligibility criteria and the treating clinician agrees that the patient may be approached, research coordinators contact them directly to provide comprehensive information about the study and answer any questions the potential participant may have. The patient is then given at least 24 hours to decide whether to partake in the study and sign the consent form, indicating their participation. The informed consent form used in the study can be accessed via the OSF repository (DOI: 10.17605/OSF.IO/CJHPV). Once patients have provided written informed consent, the baseline assessment is scheduled within 0–5 days before discharge.

### Reasons for non-participation

Reasons for non-participation could be the following: not meeting inclusion criteria, patients deemed clinically unstable, lack of interest in study participation, or concerns about the potential burden of participation. There is also a high risk of dropout due to this population often being highly burdened, the intensive assessment schedule and the post-hospital period, which represents a particularly critical phase.

### Data collection

#### Baseline assessment (T0)

The baseline assessment (T0) is conducted 0–5 days before hospital discharge by two trained research coordinators. On the day of T0, the research coordinators meet the participant on the ward and accompany them to the lab. The baseline assessment is divided into a morning and an afternoon session, separated by a break of at least 1 hour to allow the participant sufficient recovery time and minimise participant burden (see figure 1). The morning session includes the Montgomery-Åsberg Depression Rating Scale (MADRS),^29^ a single-item visual analogue scale to assess prior-night sleep quality (0, ‘very badly’—100, ‘very well’),^30^ a resting-state EEG and ECG, as well as a video-recorded verbal task with simultaneous EEG and ECG. During the afternoon session, the research coordinators assist the participant in installing the monitoring application on their personal mobile device. Next, the participant completes several self-report questionnaires, including an optional set of menstrual-health related questions (hereafter referred to as ‘Menstrual Health Questions’, MHQ) for individuals with current or past menstrual cycles (see table 1).^31^,^33^ Finally, in individuals with a history of suicide attempts, an optional interview (‘Intrusive Memory Interview’, IMI) is conducted to assess intrusive memories associated with the attempt.^34^

**Table 1:** List of self-report questionnaires

	T0	FU1	FU2
ACSS-FAD	x		
CTQ	x		
Demographics	x		
LEC-5	x		
NSSI item*	x		
SA items†	x		
SI-items‡	x		
SSS	x		
VAS-Sleep	x		
MHQ	x	x	
BDI	x	x	x
BSS	x	x	x
BHS	x	x	x
GSE	x	x	x
INQ	x	x	x
PHQ-9	x	x	x
PSQI	x	x	x
uMARS		x	
SCI§		x	x
REQ			x

* Single item: ‘In your life, have you purposefully hurt yourself without wanting to die?’ (yes/no)

† Single item: ‘How many times in your lifetime have you made an attempt to kill yourself during which you had at least some intent to die?’

‡ Four items: Suicidal ideation items: (1) ‘Currently life is not worth living’. (2) ‘Currently there are more reasons to die than to live for’. (3) ‘Currently I want to die’. (4) ‘Currently I am thinking about taking my own life’. (0/not at all–4/extremely)

§ Two items: (1) ‘Have you attempted suicide since leaving the hospital?’. If the participant indicates yes, they are asked to provide the number of attempts and the respective date(s) of the attempt(s). (2) ‘Did you need acute care since leaving the hospital? (Rehospitalisation or going to an emergency department)‘ If the participant indicates yes, they are asked to specify the type of care, as well the date and reason for hospitalisation.

ACSS-FAD, Acquired Capability for Suicide Scale–Fearlessness about Death; BDI, Beck Depression Inventory; BHS, Beck Hopelessness Scale; BSS, Beck Scale for Suicide Ideation; CTQ, Childhood Trauma Questionnaire; GSE, Generalised Self Efficacy Scale; INQ, Interpersonal Needs Questionnaire; LEC-5, Life Events Checklist; MHQ, Menstrual Health Questions; NSSI, non-suicidal self-injury, single item; PHQ-9, Patient Health Questionnaire; PSQI, Pittsburgh Sleep Quality Index; REQ, Research Experience Questionnaire; SA, suicide attempt, single item; SCI, Suicidal Crisis Information, five items; SI, suicidal ideation, four items; SSS, subjective social status; uMARS, Mobile Application Rating Scale; VAS-Sleep, Visual Analogue Sleep Scale.

MADRS: The MADRS is a well-established, clinician-administered instrument that is frequently used in clinical and research settings. It consists of 10 items, each rated on a scale from 0 to 6.^29 35^ The items assess the following dimensions: clinician-rated sadness, reported sadness; inner tension, sleep disturbances, loss of appetite, difficulties concentrating, anhedonia, emotional numbness, pessimistic thoughts and suicidal ideations. Total scores range from 0 to 60, with higher scores indicating more severe depressive symptoms. During the baseline assessment, the MADRS is administered by two research coordinators: one interviews while the other assists by taking notes and providing support as needed. After the interview, research coordinators compare scores for the individual items and agree on a consensus score for deviations greater than one for single items and deviations greater than four for overall scores. Additionally, the MADRS interview is video recorded for quality assessment and further analysis.^36^

**Figure 2:**
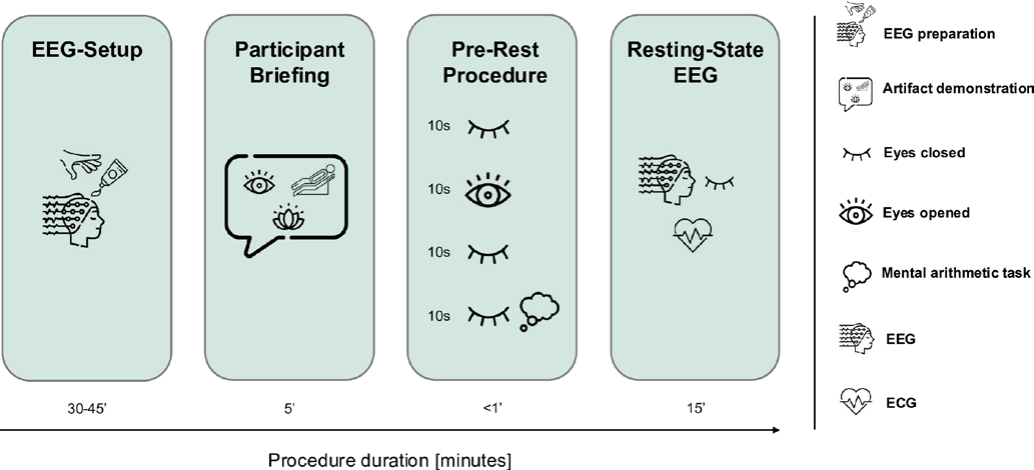
Procedure and timeline of the electroencephalogram (EEG) resting-state recording conducted during the baseline assessment. EEG setup: During the setup phase, the EEG cap is placed on participants’ heads. Afterwards, a conductive gel is applied to maintain electrode impedance below 50 kilo-ohm. Additionally, two sets of bipolar electrodes are applied to the wrists and outer canthi to record ECG and electrooculogram (EOG) activity, respectively. Participant briefing: During the briefing phase, participants receive a demonstration of common EEG artefacts (ie, eye and muscular movements) and resting-state task instructions. Prerest procedure: The prerest procedure includes four 10 s blocks: eyes closed, eyes open, eyes closed and a mental arithmetical task (ie, counting backwards from 100 in steps of 7). Resting-state EEG: 15 min eyes-closed resting-state EEG with simultaneous ECG and EOG recording.

EEG recording: The EEG recordings are conducted between 9:00 and 12:00 in a light-attenuated and sound-attenuated room where the temperature is maintained at 20°C–23°C. An ECG is recorded simultaneously. Further EEG system specifications can be found on the OSF repository (DOI: 10.17605/OSF.IO/CJHPV). Two experimental paradigms are administered during the EEG recording: A 15 min eyes-closed resting-state recording to assess neurophysiological patterns at rest linked to STB and a verbal, video-recorded task, designed to elicit markers of psychopathology across multiple channels, including physiology, facial expressions, language and vocal features. For the resting-state recording, the participant is positioned in a reclining posture. Before the recording begins, common measurement artefacts are demonstrated (muscle artefacts, eye movements), followed by a standardised procedure with brief segments of eyes-open and eyes-closed and a mental arithmetic task (see figure 2). The participant is then asked to keep their eyes closed and stay as relaxed as possible without resisting the urge to fall asleep, following standardised instructions for vigilance recordings outlined in Hegerl *et al*.^37^

**Figure 3:**
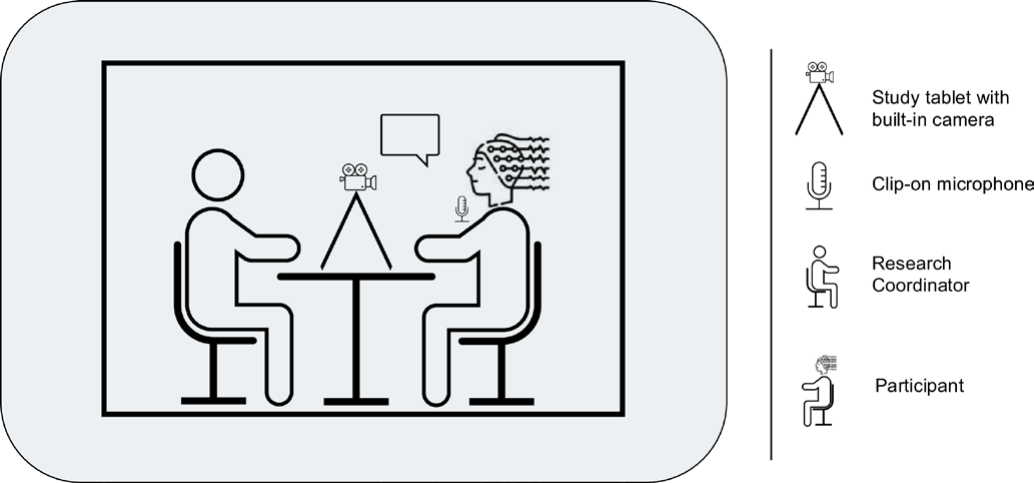
Experimental setup for the verbal task. The research coordinator is seated directly in front of the participant. A study tablet with a built-in camera is positioned on the table in front of the participant to record the task. The participant is equipped with a clip-on microphone which is connected to the laptop via a sound card. No natural light is used during the task to ensure consistent lighting conditions.

Verbal task: The verbal task is conducted in a sound- and light-shielded lab, while participants continue to wear the full EEG and ECG setup (figure 3). A research coordinator is seated directly in front of the participant, with a tablet (Dell Latitude 3140 2-in-1 laptop/tablet) positioned between them to record the task. Additionally, the participant is equipped with a microphone (RØDE Lavalier Go), which is connected to the tablet via a USB sound card (Hama 2.0 Stereo USB). To ensure consistent lighting conditions for the video recordings, no natural light is used during the verbal task. An auditory stimulus with an automated EEG trigger synchronises video and EEG recordings. The research coordinator then asks questions about experiences of different valences and time frames, resulting in four different conditions: positive/past, neutral/past, negative/past and future. This allows features, such as emotional intensity, to be compared across the four conditions. For the linguistic analysis, two additional questions are included, in which participants are asked to narrate a sequence of events and discuss the advantages and disadvantages of a topic. Hence, the task is designed to elicit extended and continuous segments of speech, which requires participants to organise information in a structured and coherent way to ensure comprehensibility. Participants have 2 min to respond to each question. To maintain standardisation, the research coordinator is generally instructed to remain silent during the 2 min response period. However, if the participant does not speak for at least five seconds, the coordinator provides one of the following prompts: ‘Can you tell me a little more about this?’ or ‘Can you explain this in more detail?’.

App installation: The monitoring smartphone application is installed on the participant’s personal mobile device with the assistance of a research coordinator. This monitoring app supports both EMA and smartphone passive sensing on Android and iOS and was developed in collaboration with the Federal Institute of Technology Zurich and Pathmate Technologies using MobileCoach (www.mobile-coach.eu), a platform for digital biomarker and health intervention research.^38 39^ During the onboarding process, a virtual coach guides participants through the process and explains the purpose of the application. Participants will be familiarised with the application, including the availability of an emergency button that offers direct contact with helpline numbers as well as contact with technical support.

Self-report questionnaires: Questionnaires are administered via the LimeSurvey platform (https://www.limesurvey.org) on a study laptop. These questionnaires assess a range of constructs, including history of self-harm, depression, hopelessness, interpersonal risk factors for STB, trauma history, self-efficacy and sleep patterns (see table 1 for a full list of questionnaires). Participants complete these independently, but a research coordinator remains nearby to answer potential questions and ensure patient safety.

IMI: The IMI^34^ is only conducted in participants with a history of suicide attempts to assess intrusive memories related to the attempt within the past 7 days. The IMI is video recorded for quality purposes.

Concerning all clinical and research-administered assessments at baseline, no digital data have yet been collected. This ensures that all ratings and observations are made independently of EMA or sensor-based information, thereby minimising potential expectancy effects and supporting unbiased measurement of participants’ initial symptomatology.

#### Mobile application data collection phase (T1)

**Figure 4:**
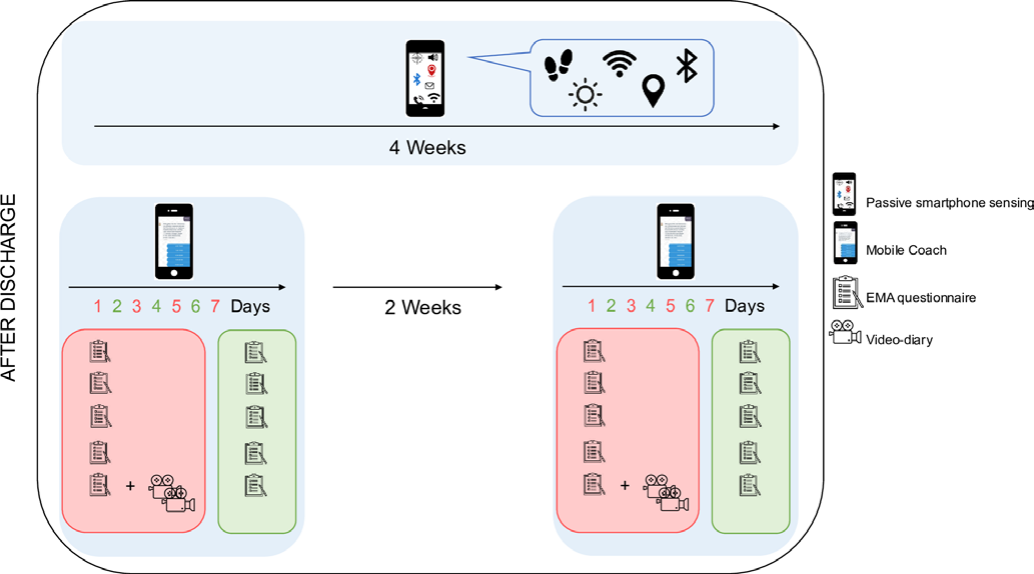
Detailed structure of the app-based data collection. Smartphone passive sensing is carried out continuously for 4 weeks via smartphone sensors, collecting data on movement, location, light and more. In addition, participants complete two active 7-day assessment periods (burst weeks) using the mobile coach app. During these bursts, participants complete five daily questionnaires and two video-diaries every other day, enabling the analysis of momentary affect, behaviour, and context. The burst weeks are scheduled in week 1 and week 4 after discharge, separated by a 2-week interval of passive monitoring only. Abbreviations: EMA, ecological momentary assessment.

The monitoring application involves active and passive collection of digital phenotyping data (see figure 4). During active data collection, the EMA protocol consists of brief self-report surveys administered five times daily across a 12-hour window (eg, 9:00 to 21:00) for seven consecutive days. This is followed by a 2-week break and a second 7-day measurement phase (see figure 4). Participants can choose their preferred 12-hour window for notifications. The EMA items assess suicide-related thoughts (passive and active ideation), mood and sleep (see the full item list on the OSF repository, DOI: 10.17605/OSF.IO/CJHPV). Additionally, every other day during the active data collection phase, participants record two 1 min video diaries in response to the following prompts: (1) ‘Can you tell us about something fulfilling that happened since the last video diary?’ and (2) ‘Can you tell us about something stressful that happened since that time?’ (see figure 4). Data are also collected passively throughout the entire 28-day period, drawing from various sensors. The availability of sensors, including, for example, bluetooth and call logs, differs depending on the phone operating system (for a complete list of all collected sensor data, see the OSF repository, DOI: 10.17605/OSF.IO/CJHPV).

Technical difficulties affecting data collections are monitored by the app (ie, missing sensor access permissions, closed app, data collection opt-out), and in case of issues, the participant is notified to actively resolve these. During the app collection phase, the coach greets users daily, encourages EMA completion and gives personalised feedback on adherence every other day. In addition to in-app notifications, reminders are sent via text message. If EMA compliance drops below 60%, a research coordinator contacts participants directly to enquire about technical difficulties or to offer additional assistance to increase compliance rates. After study completion, participants receive a personalised summary report with their analysed data on key variables such as sleep and suicidal ideation over time. Data collected with the app are securely transmitted to a server hosted by the University of Zurich (PUK and UPK site) or by NYU Langone (NYU site).

To enhance retention and reduce burden, we incorporated adherence strategies informed by a prior feasibility trial.^23^ Based on participant feedback and observed burden in that trial, we shortened the original 4-week EMA phase to two 7-day ‘boost weeks’ separated by a 2-week break. The app provides automated reminders and engagement messages, and the study coach sends daily greetings and personalised adherence feedback. Participants can select their preferred 12-hour notification window, and if EMA compliance drops below 60%, staff proactively contact them to resolve issues. To further support engagement and transparency, participants receive visual feedback at FU1 in the form of descriptive plots summarising their symptom trajectories. These measures are designed to maintain engagement and optimise data completeness.

#### Follow-up assessments (FU1 and FU2)

The first follow-up assessment (FU1) is conducted no earlier than 4 weeks and no later than 5 weeks after hospital discharge. FU1 is, by default, conducted via video chat, unless the participant prefers FU1 to be held in person, at the respective hospital. First, the participant is guided through the process of uninstalling the app. Next, the participant is asked to provide user feedback regarding the app, answering the following questions: (1) ‘How was your overall experience with our monitoring app?’, (2) ‘How did you experience answering the video diaries?’, (3) ‘How did you experience recording the video diaries?’ and (4) ‘What did you think of the two prompts for the video diaries?’. FU1 then continues with the MADRS, the verbal task and a list of self-report questionnaires (see table 1), following similar procedures as those used during the baseline assessment. Finally, each participant receives a brief personalised report summarising symptom trajectories for selected EMA items on sleep, depressive symptoms, suicidal ideation, happiness and arousal. This summary serves both as a token of appreciation for their contribution and as a source of insight into their well-being, as captured with the application during the monitoring period. Research assistants who process the EMA data for these summaries are not involved in conducting the participants’ clinical assessments.

The second follow-up assessment (FU2) is conducted 3 months after hospital discharge (±1 week). Again, FU2 is, by default, conducted via video chat, unless the participant prefers to attend in person. During FU2, the participant completes self-report questionnaires (see table 1) and receives the financial reimbursement for participating in the study. The amount of compensation is dependent on the EMA and video diary compliance, as well as the number of study visits completed. The reimbursement structure can be found on the OSF repository (DOI: 10.17605/OSF.IO/CJHPV).

Research assistants responsible for processing EMA data to generate the personalised visual summaries are not involved in conducting the participants’ clinical assessments. This separation ensures that the clinical ratings at follow-up remain fully blind to participants’ digital data, thereby minimising expectancy effects and preserving the objectivity of the assessments.

Participants undergo an extensive baseline assessment, a smartphone-based EMA phase and two follow-up appointments. To minimise burden and support well-being, assessments are designed to be brief and flexible. Participants are encouraged to pause or skip any assessment if needed. Study staff actively monitor engagement and well-being, providing guidance or support if signs of distress or fatigue are observed.

### Outcome measures

The primary outcome is self-reported suicidal ideation 4 weeks after hospital discharge, measured with the Beck Scale for Suicide Ideation (BSS).^40 41^ Additionally, we specified the following secondary outcomes:

- Self-reported suicidal ideation as measured with the BSS,^40 41^ 3 months after hospital discharge.
- Week 1 trajectory of suicidal ideation as measured with four EMA items (ie, two for active suicidal ideation and two for passive suicidal ideation).
- Week 4 trajectory of suicidal ideation as measured with four EMA items (ie, two for active suicidal ideation and two for passive suicidal ideation).
- Suicide attempts (frequency and time since discharge), assessed through participants’ self-reports and patient health records, 4 weeks and 3 months after hospital discharge.
- Hospital readmissions (frequency and duration), assessed through participants’ self-reports and patient health records, 4 weeks and 3 months after hospital discharge.
- Severity of self-reported depressive symptoms as measured with the Beck Depression Inventory,^42 43^ 4 weeks and 3 months after hospital discharge.
- Severity of investigator-rated depressive symptoms as measured with the MADRS,^29 35^ 4 weeks and 3 months after hospital discharge.

### Sample size considerations

**Figure 5:**
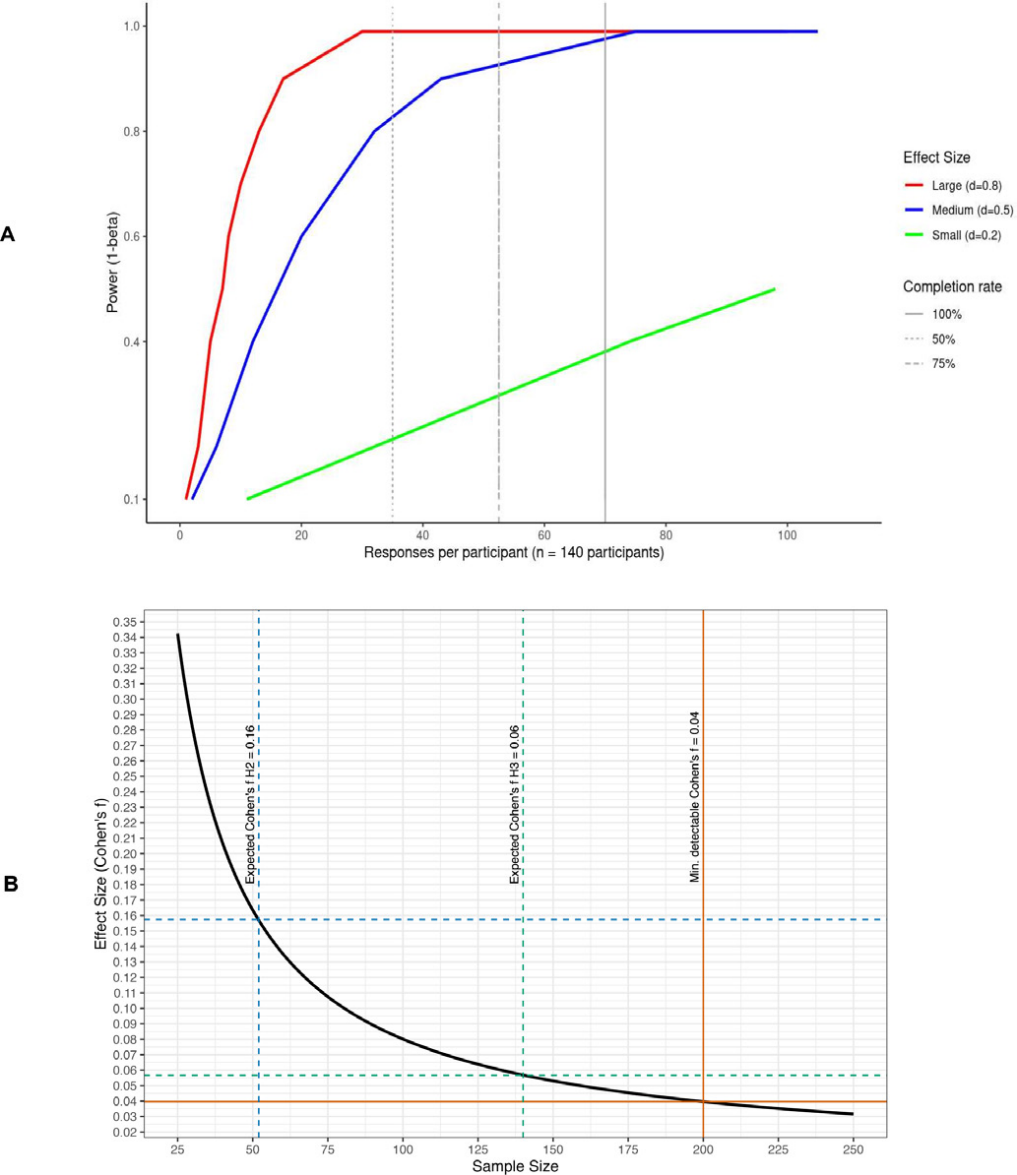
Sample size considerations for MULTICAST-PREDICT, hypotheses 1–3. The sample size calculation for EMA responses suggested that a sample size of 140 participants would be required to achieve 80% power, given a medium expected effect size and EMA completion rate of 50% (ie, 35 completed prompts per participant). We also expect a high dropout rate of 30% which leads to the final sample size of N=200 participants. (**A**) Power as a function of EMA response rate (**H1**). The plot illustrates how statistical power varies with the number of EMA responses per participant for small (d=0.2), medium (d=0.5) and large (d=0.8) effect sizes. Vertical reference lines indicate hypothetical completion rates of 50%, 75% and 100%. Given that the EMA data collection includes a total of 70 prompts, a 50% completion rate would correspond to 35 usable responses. Under this scenario, an expected medium effect size of d=0.5 would still be detectable at 80% in a sample size of N=140. (**B**) Sensitivity power analysis for H2 and H3. The plot illustrates the minimal detectable effect size (Cohen’s f) across a range of sample sizes based on a linear regression model (=0.05, power=80%). Vertical dashed lines mark the expected effect sizes derived from prior meta-analytic evidence (H2: f=0.16; H3: f=0.06), while the solid vertical line indicates the minimal detectable effect size (f=0.04) under the planned total sample size of n=200. Hence, accounting for an anticipated dropout rate of 30%, a total sample size of n=200 is sufficient to ensure adequate statistical power. Abbreviations: EMA, ecological momentary assessment; MULTICAST-PREDICT, A Multidisciplinary Approach to Predict and Treat Suicidality.

Given that MULTICAST-PREDICT was based on the SIMON study,^23^ we first calculated the required sample size based on EMA responses, using a web extension of the *R*-based *EMAtools* package.^44 45^ Based on the SIMON data, we expect a medium effect size and EMA responses of 50%.^23^ Hence, a sample size of 140 participants would be required to achieve 80% power (figure 5A). We also expect a high dropout rate of 30%, resulting in a final sample size of n=200.

We conducted a sensitivity power analysis for hypotheses 2 and 3 based on a linear regression model. Given a sample size of n=200, 80% power and an alpha-level of 5%, we would be able to detect a small, minimal detectable effect size of Cohen’s *f*=0.04 and of Cohen’s *f*=0.06 for an alpha-level of 1%, respectively. For hypothesis 2 and hypothesis 3, we performed a random-effects meta-analysis in *R* using the DerSimonian-Laird method (*metafor* package, V.4.8.0) to combine effect sizes from prior research on language-^19 46 47^ and EEG-based features.^48^,^50^ This resulted in a pooled expected effect size of Cohen’s *f*=0.16 for hypothesis 2 and Cohen’s *f*=0.06 for hypothesis 3. Accounting for an expected dropout rate of 30%, a sample size of n=150 (ie, excluding the NYU site) should be sufficiently powered for hypothesis 2, while a total sample size of n=200 should be sufficient for hypothesis 3 (see figure 5B).

### Data analysis and preprocessing

#### Hypothesis 1: EMA and passive sensing trajectories as markers of suicidal ideation

We will examine how digital phenotyping trajectories during the 4 weeks following hospital discharge relate to subsequent suicidal ideation. To this end, we will employ joint longitudinal outcome models that simultaneously estimate trajectories of suicidal ideation measured via EMA (five times daily for two 7-day periods) and the association of these trajectories with suicidal ideation assessed 4 weeks postdischarge. Passive smartphone sensing features (eg, step count, physical activity, ambient light, call frequency) will be incorporated as time-varying predictors in the longitudinal sub-model. The study site will be included as a random effect to account for heterogeneity across centres, and language will be included as a covariate or stratification factor to control for systematic differences in linguistic expression and potential cross-country variability in healthcare systems. This approach allows us to account for both within- and between-person variability, measurement error and informative missingness, while directly linking individual trajectories of EMA and sensing data to later suicidal ideation. Model fit and predictive performance will be evaluated using information criteria, variance explained and cross-validated prediction accuracy, including assessments of robustness across sites and languages. Joint longitudinal outcome models will include participants even if some EMA or passive sensing observations are missing, as these models inherently handle missingness at the observation level.^51^ ^52^ This allows the use of all available data without excluding participants for partial non-compliance. We will report the number of completed EMA assessments per participant and the extent of missing sensing data.

#### Hypothesis 2: linguistic features as markers of suicidal ideation

During the first step, syntactic patterns will be uniquely examined using the recordings of the verbal task from the two Swiss centres (PUK and UPK). Here, recordings in Swiss German, a group of vernaculars without a codified written variety and standardised orthography, will be transcribed manually, according to transcription guidelines that were designed for this task, specifically. Recordings in Standard German will be transcribed ordinarily using the standard orthography. Next, the transcriptions will be annotated automatically for parts-of-speech (PoS) using a PoS-tagger for Swiss German.^53^ Based on this PoS annotation, the mentioned syntactic features will be annotated manually in a JSON file, according to clear annotation guidelines. This task will be accomplished by two trained research coordinators under the close supervision of a PhD candidate. The recordings will be split evenly between the two annotators, with each annotator annotating about half of the recordings. The quality of each annotation will be controlled by the other annotator. In addition, the supervising PhD candidate will control randomly selected annotations and advise in case of ambiguities. From this transcribed data, we will extract scores measuring the syntactic variability and complexity of participants’ speech. These reflect how adept participants are in organising information structure. We are particularly interested in two novel, ad hoc syntactic features: ‘prefield variability’, which measures the variability of the first constituent of finite clauses; and ‘syntactic entropy’, which measures overall syntactic variance and complexity. Multiple linear regression models will then be applied to test if the syntactic scores are associated with suicidal ideation and the pre-specified secondary outcomes. To account for potential site-specific differences, the study site will be included as a random effect, while multilingualism, degree of education and native vs non-native speaker will be included as covariates to control for confounding effects. A significance level of p<0.1 will be applied. Model coefficients, standardised effect sizes, including standardised beta coefficients and partial *R^2^,* will be reported. Additionally, if syntactic patterns, derived from the manually transcribed and annotated Swiss data (ie, PUK and UPK), are shown to be consistent markers of suicidal ideation, we will also explore their cross-site scalability and examine whether these patterns can be reliably identified using LLMs, independent of the spoken language. As such, data from all three centres will undergo automatic transcription using Whisper-large-v3 (OpenAI; https://huggingface.co/openai/whisper-large-v3), which was shown to output a useful Standard German transcription to Swiss German audio,^54^ or an equivalent model, followed by proof-reading through local research coordinators.^55^ For multiple linear regression analyses, models will be estimated within a structural equation modelling framework using full-information maximum likelihood (FIML) to handle missing predictor and outcome values. FIML uses all observed data and provides unbiased estimates under a missing-at-random assumption. We will report the extent and temporal pattern of missingness, assess whether missingness relates to observed covariates, and conduct sensitivity analyses comparing FIML estimates with complete-case and multiple-imputation results.

#### Hypothesis 3: EEG resting-state signatures as markers of suicidal ideation

EEG data will be preprocessed using Python/MNE,^56^ DeepPsy Software^57^ and MATLAB/EEGlab^58^ by trained investigators. The semiautomatic preprocessing pipeline involves the following steps: (1) Identification of 15 min resting-state segment using prespecified recording markers; (2) Bandpass-filtering using finite impulse response filters with a Hann window function, including a high-pass (0.1 Hz), low-pass (70 Hz), and notch filter (50 Hz for PUK and UPK sites, 60 Hz for NYU site); (3) Manual artefact rejection of 1 s epochs which have been identified as containing movement, muscular or technical artefacts by a trained rater. Importantly, only EEG recordings with at least 80% artifact-free resting-state data will be included for further analysis; (4) Independent component analysis (FastICA algorithm) to exclude a maximum of four components which have been identified to pertain to horizontal or vertical eye movements by a trained rater; (5) Re-referencing to grand average reference and second round of manual artefact rejection. The alpha frequency range will be defined as 8–13 Hz.

Statistical analysis will be performed in *R*.^45^ Participants with missing outcome or EEG data, as well as those with less than 80% of artefact-free resting-state EEG data, will be excluded from this specific analysis. No imputation of missing data will be performed. To investigate whether baseline EEG resting-state features related to the alpha frequency range (8–13 Hz), including EEG vigilance, frontal alpha asymmetry and alpha functional connectivity, are associated with suicidal ideation after hospital discharge, we will fit a linear mixed-effects model. The study site will be modelled as a random intercept to account for between-site variability. Age, gender, baseline suicidal ideation and baseline depressive symptoms will be included as fixed-effect covariates to control for potential confounding effects. Statistical significance will be evaluated at p<0.05, and standardised fixed-effect coefficients, as well as mixed-model *R^2^* metrics, will be reported. Additionally, we will evaluate predictive model performance using leave-one-out cross-validation, reporting root mean squared error (RMSE), mean absolute error (MAE) and cross-validated *R^2^*.

#### Hypothesis 4: multimodal baseline features as predictors for suicidal ideation

To enable joint analysis of heterogeneous data sources with various temporal resolutions, we will harmonise all datasets using a standardised temporal framework anchored to the baseline, 1 month and 3-month assessment windows. From the baseline period, each modality will be processed into modality-specific embeddings or engineered features appropriate for downstream fusion. EMA responses (five prompts/day in weeks 1 and 4) and video diaries (every other day) will be summarised into windowed time-series features capturing mean levels, volatility, diurnal patterns and short-term slopes. Passive sensing streams will be converted into daily aggregates and behavioural-rhythm descriptors. These temporally dense data will be aligned to the nearest assessment window using fixed-size windows and lag variables to reflect short-term precursors of suicidal ideation. Clinical, behavioural and psychosocial questionnaires, as well as repeated video tasks and medication updates, will be processed in parallel with baseline features; temporal changes will be included as candidate predictors. We will evaluate two primary modelling architectures: (1) Early fusion—Combines modality-specific embeddings into a shared latent space and (2) Late fusion—Separate modality-specific predictors are trained independently and then integrated through a calibrated meta-learner. We will benchmark traditional machine-learning models alongside deep-learning architectures optimised for multimodal tabular and sequential data. Model training and selection will use nested, site-aware cross-validation. Learning-curve analyses will be conducted to confirm that the available sample size supports stable generalisation. To enhance clinical interpretability, we will use SHAP decompositions to quantify the contributions of each modality and each feature to model predictions. For early- and late-fusion models, missing EMA and sensing data will be handled through modality-specific preprocessing. Early-fusion models, which combine modalities into a joint feature set, will use light, modality-appropriate imputations (eg, short-gap interpolation) together with missingness indicators so the model can distinguish imputed from observed values. In late-fusion models, each modality is processed separately; missing modalities will be masked, and fusion will rely on available modality outputs only. We will document missingness patterns and examine the robustness of results to alternative preprocessing strategies.

#### Exploratory analyses

In the exploratory analyses of the smartphone data, we will extract features from smartphone sensor and EMA data, also making use of existing feature extraction frameworks (eg, the RAPIDS framework).^59^ Additionally, the data will be annotated with metadata that describes the relationships between collected variables, as specified in an application ontology. An ontology is a formal representation of knowledge that defines concepts and the relationships between them.^60^ This tool allows us to integrate heterogeneous data and to promote more data-efficient prediction of future symptom trajectories. Study site and language will be included as covariates or features in machine learning models to account for potential cross-site and cross-language variability. We will obtain EMA score predictions from machine learning models for time series forecasting, using as inputs on one hand extracted features and on the other annotated data after an embedding step, leveraging fine-tuning and zero-shot learning. Model performance will be evaluated using RMSE, MAE, mean absolute scaled error and symmetric mean absolute percentage error. In the ontology-based analyses, missing EMA and sensing data will be represented explicitly within the ontology through structured metadata that distinguishes between missing values, sensor gaps and valid zeros. This allows models to use all available information without excluding participants. When imputations are required, these will be encoded and traceable within the ontology. We will report missingness patterns and conduct sensitivity checks on alternative preprocessing strategies.

## Discussion

The period immediately following discharge from psychiatric inpatient treatment is marked by an elevated risk for suicide.^11^ However, conventional assessment approaches through infrequent clinician visits fail to capture the rapidly fluctuating nature of suicidal ideation, and current prediction models show limited utility.^5 7 12 13^ To capture the full complexity of STB, especially during this critical period, comprehensive and interdisciplinary monitoring strategies are warranted.^8 9^ Combining time-efficient ambulatory assessments, including EMA, smartphone passive sensing and digital biomarkers, with clinical, psychological, neurobiological and linguistic features assessed during the predischarge stage could be a promising approach.

Hence, the primary aims of MULTICAST-PREDICT are to investigate if and how baseline and continuously assessed measures are associated with suicidal ideation in the postdischarge period (1) as stand-alone correlates and (2) within a multimodal predictive model, applying a deep phenotyping approach to integrate multiple data sources, aligning with state-of-the-art frameworks on STB.^8 61^ While suicidal ideation assessed at a single time point is defined as the primary outcome, this project also accounts for the dynamic nature of STB by incorporating continuous ambulatory assessments as secondary outcomes. Additionally, we apply a transdiagnostic approach, including individuals with STB, ranging from passive to active suicidal ideation with or without a history of suicidal behaviour, to reflect the entire spectrum of STB and its complexity. While the combination of measurements and assessments provides rich, longitudinal data, we carefully balanced data collection with participant well-being. Clinical best practices will be followed, including limiting assessment duration, allowing flexible timing, monitoring for distress and providing feedback or support as needed, particularly given the high-risk nature of the population. Importantly, the multi-centric nature of this project will enhance the generalisability and external validity of our findings by capturing a more diverse patient population and reducing site-specific biases.

Being the first large-scale project to adopt a multidisciplinary approach to improve monitoring strategies for STB after hospital discharge, we acknowledge the exploratory nature of our endeavour. While no intervention is being tested at this stage, the study is an important step towards developing more personalised, temporally sensitive approaches to suicide risk assessment. In particular, it may yield insights into when and for whom an intervention could be most urgently needed, laying the groundwork for future just-in-time adaptive interventions.

## Ethics and dissemination

Ethical approval for the Zurich (PUK) and Basel sites (UPK) was obtained from the Ethics Committee of the Faculty of Arts and Social Sciences of the University of Zurich, Switzerland, under a joint ID (Ref: 22.09.19). The Institutional Review Board of NYU Langone Health approved the study for the New York site (NYU, Ref: i23-00366). All procedures will be conducted in accordance with the Declaration of Helsinki.^62^ Participants receive all necessary information regarding the study, including its rationale and contents, the voluntary nature of study participation, as well as data security and storage specifications, to provide written informed consent before study participation. Participants will also be informed that they may withdraw from the study at any time. Baseline and follow-up assessments will be conducted by trained investigators. In situations requiring intervention, predefined safety procedures will be followed. Additionally, contact information of emergency helplines is being provided on the patient information form and within the monitoring application, ensuring appropriate support throughout the study. Personal identifiers (eg, names, address of residency) will be removed from collected data and stored separately in a file only accessible by core members of the study team. When shared with overseas partners, the data will first be anonymised by computing the features required for the analysis. As such, only features will be shared. No encryption is performed on any stored study data. However, data will be stored securely within the local university network, and only authorised members of the study team and collaborators bound by a confidentiality agreement can access the server. Measures to protect the data against accidental or unlawful destruction are ensured by the university’s secure IT infrastructure, which includes access control based on personalised login credentials, automatic daily backups, firewall protection and continuous security monitoring by the local IT services.

To promote transparency, study materials and analysis scripts will be made publicly available on the OSF repository (DOI: 10.17605/OSF.IO/CJHPV). Additionally, in line with ethical and data governance requirements, we will share data in the form of derived features on the OSF repository wherever possible rather than raw or minimally processed data. Data will be released once collection has been completed across all three study sites. Sharing procedures will follow FAIR principles, ensuring that material will be findable, accessible, interoperable and reusable. Study findings will be primarily shared through peer-reviewed, open-access publications to enable timely and accessible communication across clinicians and the scientific community. Key findings will also be disseminated via additional formats, such as conference presentations, patient and public events, and dedicated social media outlets to reach both professional and lay audiences.
