# Supplementary material for: Deep phenotyping of suicidal ideation after discharge from psychiatric inpatient care: study protocol for an interdisciplinary, multicentre prospective observational study in Psychiatric University Hospitals
Source: BMJ Open. 2026 Feb 6;16(2):e111273. doi: 10.1136/bmjopen-2025-111273 (PMC12887521; doi:10.1136/bmjopen-2025-111273)
Supplement: Supplementary file 1 [file bmjopen-2025-111273.reviewer_comments.pdf]

PEER REVIEW HISTORY

BMJ Open publishes all reviews undertaken for accepted manuscripts. Reviewers are asked to complete a checklist review form and are provided with free text boxes to elaborate on their assessment. These free text comments are reproduced below.

ARTICLE DETAILS

Title (Provisional)

Deep Phenotyping of Suicidal Ideation After Discharge from Psychiatric Inpatient Care: Study Protocol for an Interdisciplinary, Multicentre Prospective Observational Study in Psychiatric University Hospitals

Authors

Monn, Anna; Homan, Stephanie; Mocellin, Jacopo; Raja, Schwarna Maria; Kirchhofer, Lara; Walser, Vivienne; Dolev, Eyal Liron; Nißen, Marcia; Kowatsch, Tobias; Seiler, Guido; Schultebraucks, Katharina; Olbrich, Sebastian; Kleim, Birgit

---

VERSION 1 - REVIEW

**Reviewer**	**1**
**Name**	**Steardo, Luca**
**Affiliation**	**Magna Graecia University of Catanzaro**
**Date**	**21-Oct-2025**
**COI**	**None**

This study protocol presents an ambitious and methodologically rigorous investigation into the post-discharge monitoring of suicidal ideation using a deep phenotyping approach that integrates psychological, linguistic, neurobiological, and digital data sources. The rationale for the study is strong and timely, addressing a critical gap in suicide prevention by targeting the highly vulnerable transition period following psychiatric hospitalization. The authors provide a comprehensive overview of the study design and hypotheses, and the protocol demonstrates a high level of interdisciplinarity, technical sophistication, and ethical awareness. However, several aspects could be clarified and refined to strengthen the manuscript’s transparency, interpretability, and feasibility in alignment with the expectations of BMJ Open for study protocols.

The introduction is well structured and grounded in current theoretical frameworks, particularly the Integrated Motivational–Volitional Model of Suicidal Behaviour, but the authors could more explicitly articulate how this study extends beyond previous digital phenotyping and suicide monitoring initiatives. While the integration of linguistic and EEG markers within a multimodal predictive framework is innovative, it would be useful to

delineate more clearly which elements are genuinely novel compared to previous projects such as SIMON, MOBIUS, or other post-discharge digital follow-up studies. Clarifying this distinction would help the reader understand the added scientific and translational value of the present work. The four hypotheses are well defined but would benefit from a clearer hierarchical organization to distinguish primary confirmatory objectives from exploratory or developmental ones. For instance, the fourth hypothesis, which concerns multimodal machine learning integration, appears to encompass and depend upon the preceding three; stating this relationship explicitly would improve conceptual coherence and prevent interpretive ambiguity when results are later published.

The design is described in detail, and the inclusion of multiple centers across two countries is commendable, as it enhances generalizability. Nonetheless, the manuscript should discuss in greater depth how site heterogeneity, language differences, and healthcare system variations will be statistically addressed, for example by including site as a random effect or stratifying analyses. The timeline of assessments (baseline, EMA, follow-ups) is clearly presented, but the complexity of the data collection schedule and participant burden may raise concerns about retention and data completeness. The authors mention anticipated dropout, yet further discussion of specific adherence strategies, such as automated engagement messages, clinician involvement, or interim incentives, would enhance confidence in the study's feasibility. It should also be clarified whether clinician-rated assessments such as the MADRS will be conducted blind to digital data to minimize expectancy effects.

The sample size estimation is reasonable and supported by prior work, but the derivation of the assumed effect sizes (Cohen's $f = 0.16$ and $f = 0.06$) could be better justified by referencing concrete studies from which these were drawn. Given the heterogeneity and noise typical of ecological momentary and EEG data, a sensitivity analysis exploring plausible dropout and effect size scenarios would strengthen the rationale. The planned data analyses are technically sound and modern, yet the description of the multimodal integration and machine learning components is dense and could be simplified for broader readership. The authors should specify more clearly how data from modalities with different temporal resolutions will be synchronized and weighted and whether interpretability methods such as feature importance or SHAP analyses will be used to enhance clinical meaning and transparency.

Measurement validity and data handling procedures are generally sound, but the protocol would benefit from additional operational detail. It would be helpful to specify the minimum EMA compliance required for inclusion in analyses and how missingness will be handled beyond joint models. The procedures for transcription, manual annotation, and quality control of linguistic data should describe the assessment of inter-rater reliability, while EEG preprocessing could be summarized more succinctly, including core parameters such as artifact rejection thresholds and frequency band definitions. Ethical standards are appropriately addressed, yet given the collection of video and audio data, the manuscript

should describe more explicitly the encryption, anonymization, and data storage protocols, especially in light of transatlantic data transfer. Clarifying whether participants retain the right to request deletion of their audiovisual data after study completion would further strengthen the ethical transparency.

The “Strengths and Limitations” section is concise but could be expanded to discuss potential biases introduced by digital literacy disparities, smartphone model variability, and reactivity effects from repeated EMA prompting, which may alter participants’ emotional states. It might also be worth considering how the authors will manage the balance between data richness and participant well-being, given the intensive monitoring schedule in a high-risk population. The open science approach, including preregistration and adherence to FAIR principles, is exemplary and should serve as a model for future psychiatric research; however, the authors might specify the anticipated timeline and scope of data sharing (e.g., whether de-identified processed data or only derived features will be made publicly available).

From a stylistic standpoint, the manuscript is well written and logically structured, though occasionally overly dense in the Methods section. Simplifying long sentences and maintaining consistent terminology—for example, choosing between “digital phenotyping” and “smartphone passive sensing” world improve readability. Minor grammatical refinements (e.g., replacing progressive forms such as “participants are being familiarized” with “participants will be familiarized”) would further enhance fluency.

Overall, this is an outstanding and forward-looking protocol that addresses an urgent clinical challenge with impressive methodological sophistication. The integration of multimodal data sources and advanced computational methods has the potential to transform the way suicidal ideation is monitored after discharge. I recommend acceptance pending minor revision, primarily to improve clarity regarding the analytical hierarchy, data integration strategy, participant retention and ethical safeguards for video data. With these refinements, the protocol will represent an exemplary contribution to transparent, interdisciplinary, and ethically sound psychiatric research.

VERSION 1 - AUTHOR RESPONSE

<u>In response to the reviewer, Dr. Luca Steardo:</u>

2.1 The introduction is well structured and grounded in current theoretical frameworks, particularly the Integrated Motivational–Volitional Model of Suicidal Behaviour, but the authors could more explicitly articulate how this study extends beyond previous digital phenotyping and suicide monitoring initiatives. While the integration of linguistic and EEG markers within a multimodal predictive framework is innovative, it would be useful to delineate more clearly which elements are genuinely novel compared to previous projects such as SIMON, MOBIUS, or other post-discharge digital follow-up studies. Clarifying this

distinction would help the reader understand the added scientific and translational value of the present work.

Response: Thank you for this valuable comment. We agree that it is important to emphasize how our study extends beyond previous studies, which focused on the monitoring of suicidal thoughts and behaviors after hospital discharge. While previous studies reported promising results for the predictive value of suicide-related constructs assessed via EMA, the incremental value of passive sensing data to predict short or long-term suicidal thoughts and behaviors requires further evaluation. In addition, this will be the first study to also explore the added value of linguistic and EEG resting-state features as markers of suicidal ideation after hospital discharge.

Action taken: We revised the Introduction section to more clearly highlight these unique contributions and innovative aspects of our approach.

Introduction, pp. 7 & 8

> *"Passive data collection, in contrast, occurs unobtrusively, for example via smartphone sensors (e.g., GPS, call logs, or step counts) [17]. Several studies have investigated how within- and between-person dynamics of EMA-assessed suicide-related constructs are associated with near-term and long-term STB during the post-discharge period, with some showing promising results [21–24]. Conversely, the extent to which passive data collection methods offer incremental predictive value has yet to be determined [25].*
>
> *Addressing these gaps, the present study will combine both active and passive data collection methods. It will extend beyond previous EMA-passive-sensing studies by incorporating objective pre-discharge neurobiological markers derived from resting-state electroencephalography (EEG), as well as video-based assessments to extract digital biomarkers and language-based features. Integrating these complementary data streams could represent a promising strategy for enhancing the accuracy and clinical utility of STB monitoring strategies in the post-discharge period [26]. Hence, this multicentre, observational study, MULTICAST-PREDICT (A Multidisciplinary Approach to Predict and Treat Suicidality), has two main goals: (1) to examine how key baseline variables (i.e., language- and speech-based, demographic, clinical-psychological, and neurobiological characteristics) and daily diary assessments capturing speech, clinical, and behavioural correlates are associated with suicidal ideation after hospital discharge, and (2) to study suicidal ideation in at-risk individuals using a deep-phenotyping approach, combining the available data sources."*

2.2 The four hypotheses are well defined but would benefit from a clearer hierarchical organization to distinguish primary confirmatory objectives from exploratory or developmental ones. For instance, the fourth hypothesis, which concerns multimodal machine learning integration, appears to encompass and depend upon the preceding three; stating this relationship explicitly would improve conceptual coherence and prevent interpretive ambiguity when results are later published.

Response: This is an important point. While the four hypotheses are related, they are conceptually distinct and employ different methodological approaches. Thus, hypothesis four does not depend upon the preceding three.

Action taken: To prevent ambiguity and increase coherence, we rephrased the reporting of the hypotheses as follows:

Introduction, Hypotheses, p. 8

> *"According to the main research aims, we stated the following primary hypotheses, each targeting a distinct domain or modality of investigation:*
> *(H1) Individual trajectories of digital…."*

2.3 The design is described in detail, and the inclusion of multiple centers across two countries is commendable, as it enhances generalizability. Nonetheless, the manuscript should discuss in greater depth how site heterogeneity, language differences, and healthcare system variations will be statistically addressed, for example by including site as a random effect or stratifying analyses.

Response: Thank you for raising this important point. Considering potentially confounding factors such as those is crucial for the analyses. We will expand the manuscript to clarify how site heterogeneity, language differences, and healthcare system variations are addressed analytically. Specifically, for all analyses, we plan to include the study site as a random effect in mixed-effects models to account for potential clustering and variability across centers or a covariate, where appropriate. For analyses involving language-dependent features, such as syntactic patterns (H2), we will conduct additional stratified analyses or include language as a covariate to control for systematic differences in linguistic expression. Moreover, for multimodal predictive modeling (H4), site and language will be included as features or as part of model validation procedures (e.g., cross-site validation) to ensure robustness across different healthcare contexts. These steps will be explicitly described in the revised Methods section to demonstrate how we statistically handle

multi-center and multi-country heterogeneity while investigating individual digital phenotyping trajectories (H1), syntactic markers (H2), resting-state EEG signatures (H3), and multimodal machine learning predictions (H4).

Action taken: We adapted the statistical analysis section accordingly.

Methods, Data analysis and preprocessing, p. 20-26

> *"Hypothesis 1: EMA and PS trajectories as markers of suicidal ideation*
>
> *We will examine how digital phenotyping trajectories during the four weeks following hospital discharge relate to subsequent suicidal ideation. To this end, we will employ joint longitudinal outcome models that simultaneously estimate trajectories of suicidal ideation measured via EMA (five times daily for two seven-day periods) and the association of these trajectories with suicidal ideation assessed four weeks post-discharge. Passive smartphone sensing features (e.g., step count, physical activity, ambient light, call frequency) will be incorporated as time-varying predictors in the longitudinal sub-model. The study site will be included as a random effect to account for heterogeneity across centers, and language will be included as a covariate or stratification factor to control for systematic differences in linguistic expression and potential cross-country variability in healthcare systems. This approach allows us to account for both within- and between-person variability, measurement error, and informative missingness, while directly linking individual trajectories of EMA and sensing data to later suicidal ideation. Model fit and predictive performance will be evaluated using information criteria, variance explained, and cross-validated prediction accuracy, including assessments of robustness across sites and languages….*
>
> *Hypothesis 2: Linguistic features as markers for suicidal ideation*
>
> *[…] We are particularly interested in two novel, ad hoc syntactic features: "prefield variability", which measures the variability of the first constituent of finite clauses; and "syntactic entropy", which measures overall syntactic variance and complexity. Multiple linear regression models will then be applied to test if the syntactic scores are associated with suicidal ideation and the pre-specified secondary outcomes. To account for potential site-specific differences, the study site will be included as a random effect, while multilingualism, degree of education, and native vs. non-native speaker will be included as covariates to control for confounding effects. …*

Hypothesis 3: EEG resting-state signatures as markers for suicidal ideation

[…] To investigate whether baseline EEG resting-state features related to the alpha frequency range (8-13 Hz), including EEG vigilance, frontal alpha asymmetry, and alpha functional connectivity, are associated with suicidal ideation after hospital discharge, we will fit a linear mixed-effects model. The study site will be modelled as a random intercept to account for between-site variability. Age, gender, baseline suicidal ideation, and baseline depressive symptoms will be included as fixed-effect covariates to control for potential confounding effects. Statistical significance will be evaluated at $p<0.05$, and standardized fixed-effect coefficients, as well as mixed-model R^2 metrics, will be reported. Additionally, we will evaluate predictive model performance using leave-one-out cross-validation, reporting root mean squared error (RMSE), mean absolute error (MAE), and cross-validated R^2.

Hypothesis 4: Multimodal baseline features as predictors for suicidal ideation

…We will evaluate two primary modeling architectures: (1) Early fusion - Combines modality-specific embeddings into a shared latent space, and (2) Late fusion - Separate modality-specific predictors are trained independently and then integrated through a calibrated meta-learner. We will benchmark traditional machine-learning models alongside deep-learning architectures optimized for multimodal tabular and sequential data. Model training and selection will use nested, site-aware cross-validation. Learning-curve analyses will be conducted to confirm that the available sample size supports stable generalization. To enhance clinical interpretability, we will use SHAP decompositions to quantify the contributions of each modality and each feature to model predictions.

Exploratory analyses

In the exploratory analyses of the smartphone data, we will extract features from smartphone sensor and EMA data, also making use of existing feature extraction frameworks (e.g., the RAPIDS framework) [73]. Additionally, the data will be annotated with metadata that describes the relationships between collected variables, as specified in an application ontology. An ontology is a formal representation of knowledge that defines concepts and the relationships between them. This tool allows us to integrate heterogeneous data and to promote more data-efficient prediction of future symptom trajectories [74]. Study site and language will be included as covariates or features in machine learning models to account for potential cross-site and cross-

language variability. We will obtain EMA score predictions from machine learning models for time series forecasting, using as inputs on one hand extracted features and on the other annotated data after an embedding step, leveraging fine-tuning and zero-shot learning. Model performance will be evaluated using RMSE, MAE, Mean Absolute Scaled Error (MASE), and Symmetric Mean Absolute Percentage Error (SMAPE)."

2.4 The timeline of assessments (baseline, EMA, follow-ups) is clearly presented, but the complexity of the data collection schedule and participant burden may raise concerns about retention and data completeness. The authors mention anticipated dropout, yet further discussion of specific adherence strategies, such as automated engagement messages, clinician involvement, or interim incentives, would enhance confidence in the study's feasibility.

Response: Thank you for making us aware that this point is not clearly stated yet. To support participant retention and adherence despite the intensive assessment schedule, several strategies are employed. Participants receive automated in-app engagement messages and reminders for EMA surveys and video diaries, and the study coach provides daily greetings and personalized feedback every other day. If EMA completion falls below 60%, research staff proactively contact participants to troubleshoot technical issues or provide additional support. These measures are complemented by flexible scheduling of EMA notifications, allowing participants to select their preferred 12-hour window, and by providing a personalized summary report after study completion. Together, these strategies aim to reduce participant burden, maintain engagement, and optimize data completeness.

Action taken: Therefore, we included an additional paragraph in the Methods section, detailing our approach to engage our research participants and keep compliance high.

Methods, Mobile application data collection phase (T1), p. 17

"Technical difficulties affecting data collections are monitored by the app (i.e., missing sensor access permissions, closed app, data collection opt-out), and in case of issues, the participant is notified to actively resolve these. During the app collection phase, the coach greets users daily, encourages EMA completion, and gives personalized feedback on adherence every other day. In addition to in-app notifications, reminders are sent via text message. If EMA compliance drops below 60%, a research coordinator contacts participants directly to enquire about technical difficulties or to offer additional assistance to increase compliance rates. After study completion, participants receive a personalized summary report with their analysed data on key

variables such as sleep and suicidal ideation over time. Data collected with the app are securely transmitted to a server hosted by the University of Zurich (PUK and UPK site) or by NYU Langone (NYU site).

To enhance retention and reduce burden, we incorporated adherence strategies informed by a prior feasibility trial (Sels et al., 2021; Homan et al., 2025). Based on participant feedback and observed burden in that trial, we shortened the original 4-week EMA phase to two 7-day "boost weeks" separated by a 2-week break. The app provides automated reminders and engagement messages, and the study coach sends daily greetings and personalized adherence feedback. Participants can select their preferred 12-hour notification window, and if EMA compliance drops below 60%, staff proactively contact them to resolve issues. To further support engagement and transparency, participants receive visual feedback at FU1 in the form of descriptive plots summarizing their symptom trajectories. These measures are designed to maintain engagement and optimize data completeness."

2.5 It should also be clarified whether clinician-rated assessments such as the MADRS will be conducted blind to digital data to minimize expectancy effects.

Response: We thank the reviewer for this comment. At baseline, no digital data have yet been collected; therefore, expectancy effects cannot influence the clinician-rated assessments. At follow-up, when digital data are available, all clinician-rated assessments (e.g., MADRS) are conducted by assessors who are fully blind to participants' digital data, thereby minimizing expectancy effects and ensuring unbiased ratings. The EMA responses used to generate visual feedback for participants are processed exclusively by research assistants who are not involved in conducting that participant's clinical assessments.

Action taken: To give more information about the conduction of the assessments and potentially influencing effects, we adapted the Methods as follows:

Methods, Baseline assessment, p. 15

"Intrusive Memory Interview. The IMI [40] is only conducted in participants with a history of suicide attempts to assess intrusive memories related to the attempt within the past 7 days. The IMI is video recorded for quality purposes.

Concerning all clinical and research-administered assessments at baseline, no digital data have yet been collected. This ensures that all ratings and observations are made

independently of EMA or sensor-based information, thereby minimizing potential expectancy effects and supporting unbiased measurement of participants' initial symptomatology."

Methods, Follow-up assessments (FU1 & FU2), p. 18

"FU1 then continues with the MADRS, the verbal task, and a list of self-report questionnaires (see table 1), following similar procedures as those used during the baseline assessment. Finally, each participant receives a brief personalized report summarizing symptom trajectories for selected EMA items on sleep, depressive symptoms, suicidal ideation, happiness, and arousal. This summary serves both as a token of appreciation for their contribution and as a source of insight into their well-being, as captured with the application during the monitoring period.

Research assistants responsible for processing EMA data to generate the personalized visual summaries are not involved in conducting the participants' clinical assessments. This separation ensures that the clinical ratings at follow-up remain fully blind to participants' digital data, thereby minimizing expectancy effects and preserving the objectivity of the assessments."

2.6 The sample size estimation is reasonable and supported by prior work, but the derivation of the assumed effect sizes (Cohen's f = 0.16 and f = 0.06) could be better justified by referencing concrete studies from which these were drawn.

Response: Thank you for pointing this out! We agree that the clarity of our sample size considerations could be improved by referencing the specific studies relevant to each hypothesis.

Action taken: We added the respective references accordingly.

Methods, Sampple size considerations, p. 20

"For hypothesis 2 and hypothesis 3, we performed a random-effects meta-analysis in R using the DerSimonian-Laird method (metafor package, v4.8.0) to combine effect sizes from prior research on language- [19,48,49] and EEG-based features [23,24,26]."

2.7 Given the heterogeneity and noise typical of ecological momentary and EEG data, a sensitivity analysis exploring plausible dropout and effect size scenarios would strengthen the rationale.

Response: Thank you for this thoughtful suggestion and the opportunity to clarify this. We agree that addressing heterogeneity and potential dropout is important, especially considering potential missingness due to noisy EMA and EEG data. Figure 5A) of our manuscript depicts effect size scenarios for different EMA response rates (H1), while Figure 5B) shows the results of our sensitivity power analysis showing how potential sample size scenarios would affect the minimal detectable effect size.

Action taken: To enhance clarity, we have expanded the description of these plots in the revised version to better highlight how they address dropout scenarios.

> *Figure 5*
>
> *"Sample size considerations for MULTICAST-PREDICT, hypotheses 1-3. The sample size calculation for EMA responses suggested that a sample size of 140 participants would be required to achieve 80% power, given a medium expected effect size and EMA completion rate of 50% (i.e., 35 completed prompts per participant). We also expect a high dropout rate of 30% which leads to the final sample size of N=200 participants. (A) Power as a function of EMA response rate (H1). The plot illustrates how statistical power varies with the number of EMA responses per participant for small (d = 0.2), medium (d = 0.5), and large (d = 0.8) effect sizes. Vertical reference lines indicate hypothetical completion rates of 50%, 75%, and 100%. Given that the EMA data collection includes a total of 70 prompts, a 50% completion rate would correspond to 35 usable responses. Under this scenario, an expected medium effect size of d = 0.5 would still be detectable at 80% in a sample size of N = 140. (B) Sensitivity power analysis for H2 and H3. The plot illustrates the minimal detectable effect size (Cohen's f) across a range of sample sizes based on a linear regression model (alpha = .05, power = 80%). Vertical dashed lines mark the expected effect sizes derived from prior meta-analytic evidence (H2: f = 0.16; H3: f = 0.06), while the solid vertical line indicates the minimal detectable effect size (f = 0.04) under the planned total sample size of N = 200. Hence, accounting for an anticipated dropout rate of 30%, a total sample size of N = 200 is sufficient to ensure adequate statistical power."*

2.8 The planned data analyses are technically sound and modern, yet the description of the multimodal integration and machine learning components is dense and could be simplified for broader readership. The authors should specify more clearly how data from modalities with different temporal resolutions will be synchronized and weighted and whether interpretability methods such as feature importance or SHAP analyses will be used to enhance clinical meaning and transparency.

Response: Thank you for your comment. We agree that providing a description that addresses a broad readership is important, and we revised the manuscript to simplify the description for better readability. Further, we provided more detail on how data from modalities with different temporal resolutions are synchronized and weighted, and we clarified our planned use of interpretability methods, such as feature importance and SHAP analyses, to enhance clinical insight and transparency.

Action taken: The adapted paragraph reads as follows:

> *Methods, Data analysis and preprocessing, pp. 24 & 25*
>
> *"Hypothesis 4: Multimodal baseline features as predictors for suicidal ideation*
>
> *To enable joint analysis of heterogeneous data sources with various temporal resolutions, we will harmonize all datasets using a standardized temporal framework anchored to the baseline, 1-month, and 3-month assessment windows. From the baseline period, each modality will be processed into modality-specific embeddings or engineered features appropriate for downstream fusion. EMA responses (five prompts/day in Weeks 1 and 4) and video diaries (every other day) will be summarized into windowed time-series features capturing mean levels, volatility, diurnal patterns, and short-term slopes. Passive sensing streams will be converted into daily aggregates and behavioral-rhythm descriptors. These temporally dense data will be aligned to the nearest assessment window using fixed-size windows and lag variables to reflect short-term precursors of suicidal ideation. Clinical, behavioral, and psychosocial questionnaires, as well as repeated video tasks and medication updates, will be processed in parallel with baseline features; temporal changes will be included as candidate predictors. We will evaluate two primary modeling architectures: (1) Early fusion - Combines modality-specific embeddings into a shared latent space, and (2) Late fusion - Separate modality-specific predictors are trained independently and then integrated through a calibrated meta-*

learner. We will benchmark traditional machine-learning models alongside architectures optimized for multimodal tabular and sequential data. Model training and selection will use nested, site-aware cross-validation. Learning-curve analysis will be conducted to confirm that available sample size supports stable generalization. To enhance clinical interpretability, we will use SHAP decompositions to quantify the contributions of each modality and each feature to model predictions."

2.9 Measurement validity and data handling procedures are generally sound, but the protocol would benefit from additional operational detail. It would be helpful to specify the minimum EMA compliance required for inclusion in analyses and how missingness will be handled beyond joint models.

Response: Thank you for this comment. We agree that additional operational detail on EMA compliance and handling of missing data would be valuable. Since the approach of missing data handling might vary depending on the hypothesis and statistical approach, we comment on this issue for each hypothesis separately that incorporates the use of EMA data, except for hypothesis 3, where we already stated that we will not include participants with missing data.

Action taken: Accordingly, we added this information to the statistical approach section as follows:

Methods, Data analysis and preprocessing, pp. 20-26

"Hypothesis 1: EMA and PS trajectories as markers of suicidal ideation

We will examine how digital phenotyping trajectories during the four weeks following hospital discharge relate to subsequent suicidal ideation. To this end, we will employ joint longitudinal outcome models that simultaneously estimate trajectories of suicidal ideation measured via EMA (five times daily for two seven-day periods) and the association of these trajectories with suicidal ideation assessed four weeks post-discharge. Passive smartphone sensing features (e.g., step count, physical activity, ambient light, call frequency) will be incorporated as time-varying predictors in the longitudinal sub-model. Study site will be included as a random effect to account for heterogeneity across centers, and language will be included as a covariate or stratification factor to control for systematic differences in linguistic expression and potential cross-country variability in healthcare systems. This approach allows us to account for both within- and between-person variability, measurement error, and informative missingness, while directly linking individual trajectories of EMA and

sensing data to later suicidal ideation. Model fit and predictive performance will be evaluated using information criteria, variance explained, and cross-validated prediction accuracy, including assessments of robustness across sites and languages. Joint longitudinal outcome models will include participants even if some EMA or passive sensing observations are missing, as these models inherently handle missingness at the observation level (e.g., Hedeker & Gibbons, 2006; Enders, 2010). This allows the use of all available data without excluding participants for partial noncompliance. We will report the number of completed EMA assessments per participant and the extent of missing sensing data.

Hypothesis 2: Linguistic features as markers for suicidal ideation

[..] As such, data from, which was shown to output a useful Standard German transcription to Swiss German audio (Dolev et al., 2024), centres will undergo automatic transcription using Whisper-large-v3 (OpenAI; https://huggingface.co/openai/whisper-large-v3) or an equivalent model, followed by proof-reading through local research coordinators [see also 53]. For multiple linear regression analyses, models will be estimated within a structural equation modelling framework using full-information maximum likelihood (FIML) to handle missing predictor and outcome values. FIML uses all observed data and provides unbiased estimates under a Missing-At-Random assumption. We will report the extent and temporal pattern of missingness, assess whether missingness relates to observed covariates, and conduct sensitivity analyses comparing FIML estimates with complete-case and multiple-imputation results.

Hypothesis 3: EEG resting-state signatures as markers for suicidal ideation

[…]

Hypothesis 4: Multimodal baseline features as predictors for suicidal ideation

[..] To enhance clinical interpretability, we will use SHAP decompositions to quantify the contributions of each modality and each feature to model predictions. For early- and late-fusion models, missing EMA and sensing data will be handled through modality-specific preprocessing. Early-fusion models, which combine modalities into a joint feature set, will use light, modality-appropriate imputations (e.g., short-gap interpolation) together with missingness indicators so the model can distinguish imputed from observed values. In late-fusion models, each modality is processed

separately; missing modalities will be masked, and fusion will rely on available modality outputs only. We will document missingness patterns and examine the robustness of results to alternative preprocessing strategies.

Exploratory analyses

In the exploratory analyses of the smartphone data, we will extract features from smartphone sensor and EMA data, also making use of existing feature extraction frameworks (e.g., the RAPIDS framework) [73]. Additionally, the data will be annotated with metadata that describes the relationships between collected variables, as specified in an application ontology. An ontology is a formal representation of knowledge that defines concepts and the relationships between them. This tool allows us to integrate heterogeneous data and to promote more data-efficient prediction of future symptom trajectories [74]. Study site and language will be included as covariates or features in machine learning models to account for potential cross-site and cross-language variability. We will obtain EMA score predictions from machine learning models for time series forecasting, using as inputs on one hand extracted features and on the other annotated data after an embedding step, leveraging fine-tuning and zero-shot learning. Model performance will be evaluated using RMSE, MAE, Mean Absolute Scaled Error (MASE), and Symmetric Mean Absolute Percentage Error (SMAPE). In the ontology-based analyses, missing EMA and sensing data will be represented explicitly within the ontology through structured metadata that distinguishes between missing values, sensor gaps, and valid zeros. This allows models to use all available information without excluding participants. When imputations are required, these will be encoded and traceable within the ontology. We will report missingness patterns and conduct sensitivity checks on alternative preprocessing strategies."

2.10 The procedures for transcription, manual annotation, and quality control of linguistic data should describe the assessment of inter-rater reliability, while EEG preprocessing could be summarized more succinctly, including core parameters such as artifact rejection thresholds and frequency band definitions.

Response: Thank you for this helpful comment. We completely agree that the transcription and preprocessing steps for the linguistic and EEG data, respectively, require further clarification. Analyses of syntactic patterns, which are unique to our study and require manual transcription and in-depth annotation, will be conducted exclusively on data from the two Swiss centres (University Hospital of Psychiatry Zurich and the University Psychiatric Clinics Basel). We added a detailed description of these transcription and annotation procedures to the methods section and explained

how this affects sample size considerations. In addition, we will explore the scalability of these candidate markers across centres, using automatic transcription pipelines. The preprocessing pipeline for the EEG will be applied across all three centres.

Action taken: Accordingly, we added a more detailed description of the transcription and annotation procedures to the Methods section. In addition, we included a definition of the EEG preprocessing pipeline, listing the key parameters.

Methods, Data analysis and preprocessing, pp. 21 & 22

> *" Hypothesis 2: Linguistic features as markers of suicidal ideation*
>
> *During a first step, syntactic patterns will be uniquely examined using the recordings of the verbal task from the two Swiss centres (PUK and UPK). Here, recordings in Swiss German, a group of vernaculars without a codified written variety and standardized orthography, will be transcribed manually, according to transcription guidelines that were designed for this task, specifically. Recordings in Standard German will be transcribed ordinarily using the standard orthography. Next, the transcriptions will be annotated automatically for parts-of-speech (PoS) using a PoS-tagger for Swiss German [52]. Based on this PoS annotation, the mentioned syntactic features will be annotated manually in a JSON file, according to clear annotation guidelines. This task will be accomplished by two trained research coordinators, under close supervision of a PhD candidate. The recordings will be split evenly between the two annotators, with each annotator annotating about half of the recordings. The quality of each annotation will be controlled by the other annotator. In addition, the supervising PhD candidate will control randomly selected annotations and advise in case of ambiguities. From this transcribed data, we will extract scores measuring the syntactic variability and complexity of participants' speech. These reflect how adept participants are in organizing information structure. We are particularly interested in two novel, ad hoc syntactic features: "prefield variability", which measures the variability of the first constituent of finite clauses; and "syntactic entropy", which measures overall syntactic variance and complexity. Multiple linear regression models will then be applied to test if the syntactic scores are associated with suicidal ideation and the pre-specified secondary outcomes. To account for potential site-specific differences, the study site will be included as a random effect, while multilingualism, degree of education, and native vs. non-native speaker will be included as covariates to control for confounding effects. A significance level of $p<0.1$ will be applied. Model coefficients, standardized effect sizes, including standardized beta coefficients and*

partial R2, will be reported. Additionally, if syntactic patterns, derived from the manually transcribed and annotated Swiss data (i.e., PUK and UPK), are shown to be consistent markers of suicidal ideation, we will also explore their cross-site scalability and examine whether these patterns can be reliably identified using LLMs, independent of the spoken language. As such, data from all three centres will undergo automatic transcription using Whisper-large-v3 (OpenAI; https://huggingface.co/openai/whisper-large-v3), which was shown to output a useful Standard German transcription to Swiss German audio (Dolev et al., 2024), or an equivalent model, followed by proof-reading through local research coordinators [see also 53]. […]

Hypothesis 3: EEG resting-state signatures as markers for of suicidal ideation

EEG data will be preprocessed using Python/MNE [52], DeepPsy Software [53], and MATLAB/EEGlab [54] by trained investigators. The semi-automatic preprocessing pipeline involves the following steps: 1) Identification of 15-minute resting-state segment using pre-specified recording markers; 2) Bandpass-filtering using finite impulse response filters with a Hann window function, including a high-pass (0.1 Hz), low-pass (70 Hz), and notch filter (50Hz for PUK and UPK sites, 60 Hz for NYU site); 3) Manual artefact rejection of 1-second epochs which have been identified as containing movement, muscular, or technical artefacts by a trained rater. Importantly, only EEG recordings with at least 80% artifact-free resting-state data will be included for further analysis; 4) Independent component analysis (FastICA algorithm) to exclude a maximum of four components which have been identified to pertain to horizontal or vertical eye movements by a trained rater; 5) Re-referencing to grand average reference and second round of manual artefact rejection. The alpha frequency range will be defined as 8-13 Hz.

Statistical analysis will be performed in R [46]. Participants with missing outcome or EEG data will be excluded from this specific analysis. To investigate whether baseline EEG resting-state features related to the alpha frequency range (8-13 Hz), including EEG vigilance, frontal alpha asymmetry, and alpha functional connectivity, are associated with suicidal ideation after hospital discharge, we will conduct multiple linear regression analyses."

Methods, Sample size considerations, p. 20

"For hypothesis 2 and hypothesis 3, we performed a random-effects meta-analysis in R using the DerSimonian-Laird method (metafor package, v4.8.0) to combine effect sizes from prior research on language- [19,47,48] and EEG-based features [49–51]. This resulted in a pooled expected effect size of Cohen's f = 0.16 for hypothesis 2 and

Cohen's f = 0.06 for hypothesis 3. Accounting for an expected dropout rate of 30%, a sample size of N=150 (i.e., excluding the NYU site) should be sufficiently powered for hypothesis 2, while a total sample size of N=200 should be sufficient for hypothesis 3 (see figure 5B)."

2.11 Ethical standards are appropriately addressed, yet given the collection of video and audio data, the manuscript should describe more explicitly the encryption, anonymization, and data storage protocols, especially in light of transatlantic data transfer. Clarifying whether participants retain the right to request deletion of their audiovisual data after study completion would further strengthen the ethical transparency.

Response: Thank you for this important comment. We have now expanded the section on Ethics and Dissemination to more explicitly describe our de-identification and anonymization protocols as well as data storage and cross-centre sharing procedures.

Action: Accordingly, we adapted the respective section as follows:

Ethics and Dissemination, p. 29

"Personal identifiers (e.g., names, address of residency, etc.) will be removed from collected data and stored separately in a file only accessible by core members of the study team. When shared with overseas partners, the data will first be anonymized by computing the features required for the analysis. As such, only features will be shared. No encryption is performed on any stored study data. However, data will be stored securely within the local university network, and only authorized members of the study team and collaborators bound by a confidentiality agreement can access the server. Measures to protect the data against accidental or unlawful destruction are ensured by the university's secure IT infrastructure, which includes access control based on personalized login credentials, automatic daily backups, firewall protection, and continuous security monitoring by the local IT services."

2.12 The "Strengths and Limitations" section is concise but could be expanded to discuss potential biases introduced by digital literacy disparities, smartphone model variability, and reactivity effects from repeated EMA prompting, which may alter participants' emotional states.

Response: These are great additions. Thank you for making us aware.

Action taken: We adapted the summary "Strengths and Limitations" accordingly:

Strengths and Limitations, p. 5

- *This study's strength lies in the integration of diverse data sources (psychological, linguistic, neurobiological, and digital) for deep phenotyping.*
- *The high-frequency, real-time assessments (ecological momentary assessments and video diaries) allow for capturing suicidal ideation dynamics.*
- *Potential limitations related to the study include that intensive assessments may lead to dropout or reactivity effects, as well as biases due to variability in participants' digital literacy or smartphone models.*
- *Potential limitations related to study design include the observational nature, which limits causal inference regarding risk factors for suicidal ideation.*

2.13 It might also be worth considering how the authors will manage the balance between data richness and participant well-being, given the intensive monitoring schedule in a high-risk population.

Response: This is an important point considering the vulnerable population we are studying and the potential burden introduced by the measurements and assessments.

Action taken: Therefore, we added respective considerations to the Methods and Discussion sections.

Methods, Data collection, p. 18

> *"The second follow-up assessment (FU2) is conducted three months after hospital discharge (+/ – 1 week). Again, FU2 is, by default, conducted via video chat, unless the participant prefers to attend in person. During FU2, the participant completes self-report questionnaires (see table 1) and receives the financial reimbursement for participating in the study. The amount of compensation is dependent on the EMA and video diary compliance, as well as the number of study visits completed. The reimbursement structure can be found on the repository (DOI: 10.17605/ OSF.IO/CJHPV).*

Participants undergo an extensive baseline assessment, a smartphone-based EMA phase, and two follow-up appointments. To minimize burden and support well-being, assessments are designed to be brief and flexible. Participants are encouraged to pause or skip any assessment if needed. Study staff actively monitor engagement and well-being, providing guidance or support if signs of distress or fatigue are observed."

Discussion, p. 27

"Hence, the primary aims of MULTICAST-PREDICT are to investigate if and how baseline and continuously-assessed measures are associated with suicidal ideation in the post-discharge period (1) as stand-alone correlates and (2) within a multi-modal predictive model, applying a deep phenotyping approach to integrate multiple data sources, aligning with state-of-the-art frameworks on STB [8,57]. While suicidal ideation assessed at a single time point is defined as the primary outcome, this project also accounts for the dynamic nature of STB by incorporating continuous ambulatory assessments as secondary outcomes. Additionally, we apply a transdiagnostic approach, including individuals with STB, ranging from passive to active suicidal ideation with or without a history of suicidal behaviour, to reflect the entire spectrum of STB and its complexity. While the combination of measurements and assessments provides rich, longitudinal data, we carefully balanced data collection with participant well-being. Clinical best practices will be followed, including limiting assessment duration, allowing flexible timing, monitoring for distress, and providing feedback or support as needed, particularly given the high-risk nature of the population. Importantly, the multi-centric nature of this project will enhance the generalizability and external validity of our findings by capturing a more diverse patient population and reducing site-specific biases."

2.13 The open science approach, including preregistration and adherence to FAIR principles, is exemplary and should serve as a model for future psychiatric research; however, the authors might specify the anticipated timeline and scope of data sharing (e.g., whether de-identified processed data or only derived features will be made publicly available).

Response: Thank you for this positive and constructive comment. We fully agree that transparency regarding data-sharing plans is important. In the revised manuscript, we now clarify that data will be shared once data collection has been completed across all three study sites. In line with privacy and governance requirements, we will make derived features publicly available wherever possible rather than raw or minimally processed data. All shared materials will follow FAIR to facilitate reuse.

Action taken: We added this additional information to the Ethics and Dissemination section.

Ethics and Dissemination, p. 29

> *"To promote transparency, study materials and analysis scripts will be made publicly available on the OSF repository (DOI: 10.17605/OSF.IO/CJHPV). Additionally, in line with ethical and data governance requirements, we will share data in the form of derived features on the OSF repository wherever possible rather than raw or minimally processed data. Data will be released once collection has been completed across all three study sites. Sharing procedures will follow FAIR principles, ensuring that material will be findable, accessible, interoperable, and reusable. Study findings will be primarily shared through peer-reviewed, open-access publications to enable timely and accessible communication across clinicians and the scientific community. Key findings will also be disseminated via additional formats, such as conference presentations, patient and public events, and dedicated social media outlets to reach both professional and lay audiences."*

2.14 From a stylistic standpoint, the manuscript is well written and logically structured, though occasionally overly dense in the Methods section. Simplifying long sentences and maintaining consistent terminology—for example, choosing between "digital phenotyping" and "smartphone passive sensing" would improve readability.

Response: We appreciate the reviewer's suggestion regarding terminology. While related, these terms refer to distinct constructs in our study: "digital phenotyping" captures the broader framework of using different digital data streams to characterize human behavior and health, such as passive sensing data and EMA data, whereas "smartphone passive sensing" refers specifically to the sensor-based data collected unobtrusively via participants' smartphones. Yet, we agree that consistent and well-defined terminology, as well as concise reporting important.

Action taken: First, we better defined the terminology.

Introduction, pp. 6 & 7

> *"To address this challenge, recent advancements in digital phenotyping – defined as "the moment-by-moment quantification of the individual-level human phenotype in situ using data from personal digital devices" [15] such as passive sensing data and data from ecological momentary assessments (EMA) – offer promising opportunities for more continuous and ecologically valid monitoring of suicidal ideation [16,17]. These techniques may draw from various data sources (e.g., behavioural, psychological,*

environmental, and social phenotyping) and collection methods, which are typically divided into active and passive data collection methods. Active data collection requires active user input, for example, in the form of EMA, where suicidal ideation and related constructs are assessed via repeated smartphone prompts throughout the day, or through video diaries, from which facial, speech, and linguistic features can be derived [13,17–20].”

Second, we made adaptations throughout the manuscript for a more concise reporting that would improve readability.

e.g., Methods, Data analysis and preprocessing, pp. 20-26

> *“To enable joint analysis of heterogeneous data sources with various temporal resolutions, we will harmonize all datasets using a standardized temporal framework anchored to the baseline, 1-month, and 3-month assessment windows.”*
>
> *“During the first step, syntactic patterns will be uniquely examined using the recordings of the verbal task from the two Swiss centres (PUK and UPK).”*

2.15 Minor grammatical refinements (e.g., replacing progressive forms such as “participants are being familiarized” with “participants will be familiarized”) would further enhance fluency.

Response: Thank you for making us aware.

Action taken: We corrected the manuscript for these minor grammatical mistakes, e.g.,

> *“Participants will be familiarized with the application…” (p. 15)*
>
> *“All procedures will be conducted in accordance with…” (p. 27)*
